# Supplementary material for: Genetic architecture and mechanisms of host-microbiome interactions from a multi-cohort analysis of outbred laboratory rats
Source: Nat Commun. 2025 Dec 18;16:10126. doi: 10.1038/s41467-025-66105-z (PMC12715198; doi:10.1038/s41467-025-66105-z)
Supplement: Supplementary file 1 — Supplementary Information [file 41467_2025_66105_MOESM1_ESM.pdf]

Supplementary figures and tables for:

Genetic architecture and mechanisms of host/microbiome interactions from a multi-cohort analysis of outbred laboratory rats

Helene Tonnele[1,2#], Denghui Chen[3#], Felipe Morillo[1,2], Jorge Garcia-Calleja[4], Apurva S Chitre[3], Benjamin B Johnson[3], Thiago Missfeldt Sanches[3], Riyan Cheng[3], Marc Jan Bonder[5], Antonio Gonzalez[6], Tomasz Kosciolek[6], Anthony M George[7], Wenyan Han[8], Katie Holl[9], Aidan Horvath[10], Keita Ishiwari[7,11], Christopher P King[12], Alexander C Lamparelli[12], Connor D Martin[7,11], Angel Garcia Martinez[8], Alesa H Netzley[10], Jordan A Tripi[12], Tengfei Wang[8], Elena Bosch[4], Peter A Doris[13], Oliver Stegle[14], Hao Chen[8], Shelly B. Flagel[15,16], Paul J Meyer[12], Jerry B Richards[7,11], Terry E. Robinson[10], Leah C Solberg Woods[17], Oksana Polesskaya[3], Rob Knight[6,18,19,20,21], Abraham A Palmer[3,22*], Amelie Baud[1,2*]

[#] these authors contributed equally: Helene Tonnele and Denghui Chen
* these authors are corresponding authors: Abraham A Palmer and Amelie Baud

Corresponding authors:
Abraham A Palmer: aap@ucsd.edu
Amelie Baud: amelie.baud@crg.eu

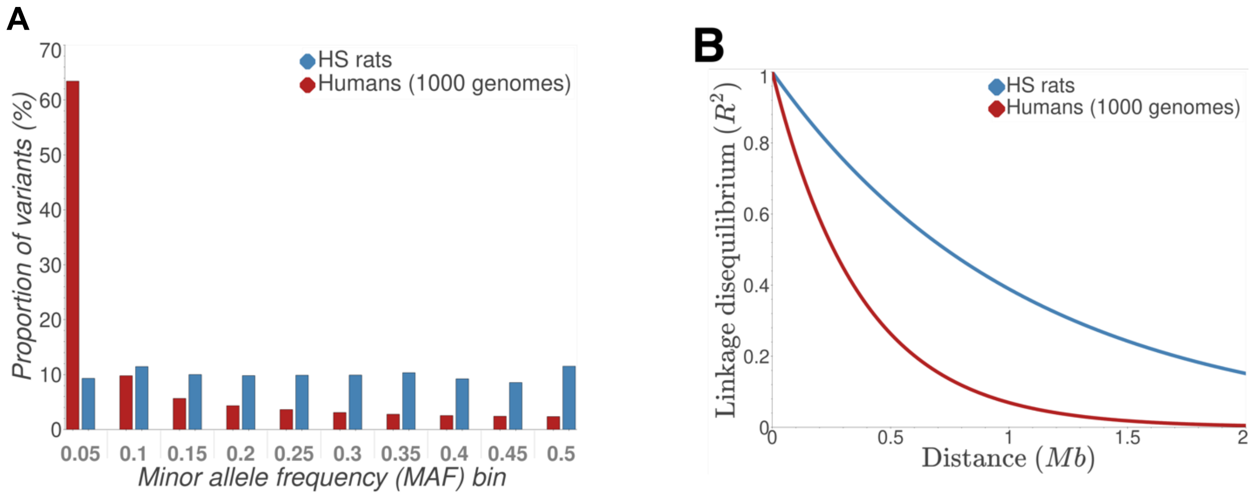

Supplementary Figure 1 Genetic characteristics of the HS population favourable for GWAS. (A) Comparison of the minor allele frequency (MAF) of genetic variants segregating in the HS rats and in humans. (B) Decay of linkage disequilibrium between neighbouring variants in the HS rats and in humans.

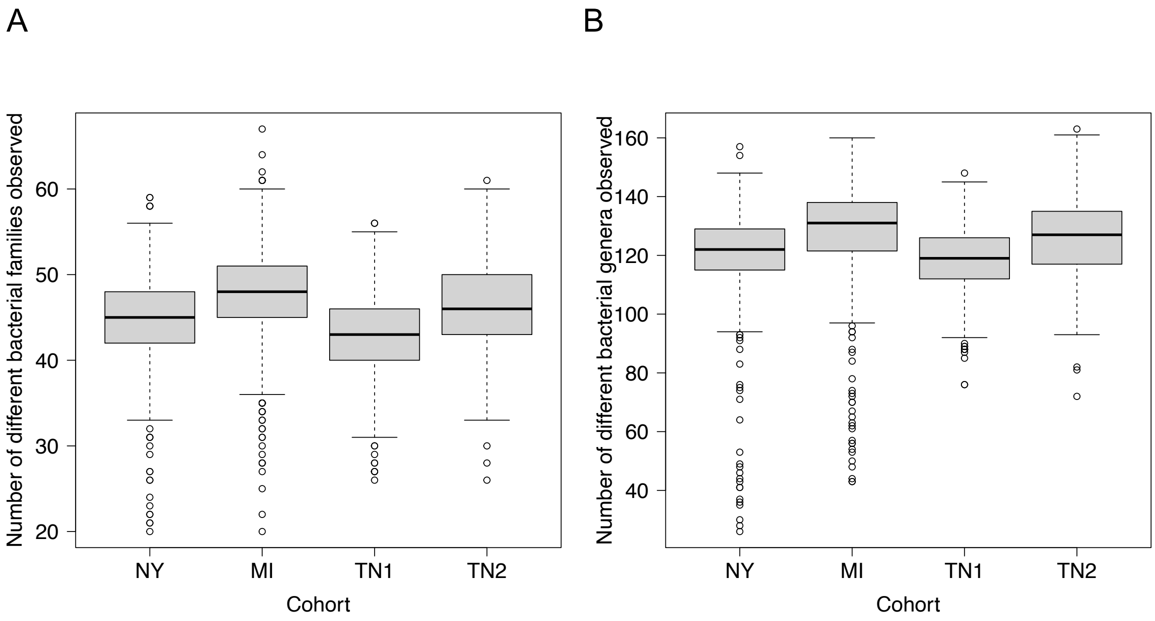

Supplementary Figure 2 Within-sample (alpha) microbiome diversity. Number of different (A) families or (B) genera observed in a sample (i.e. richness) in the different cohorts. A rarefied count table was used for this analysis (see Methods; after keeping only those rats with at least 10,000 reads and cohort information, N = 812, 763, 685 and 450 for NY, MI, TN1, and TM2 respectively).

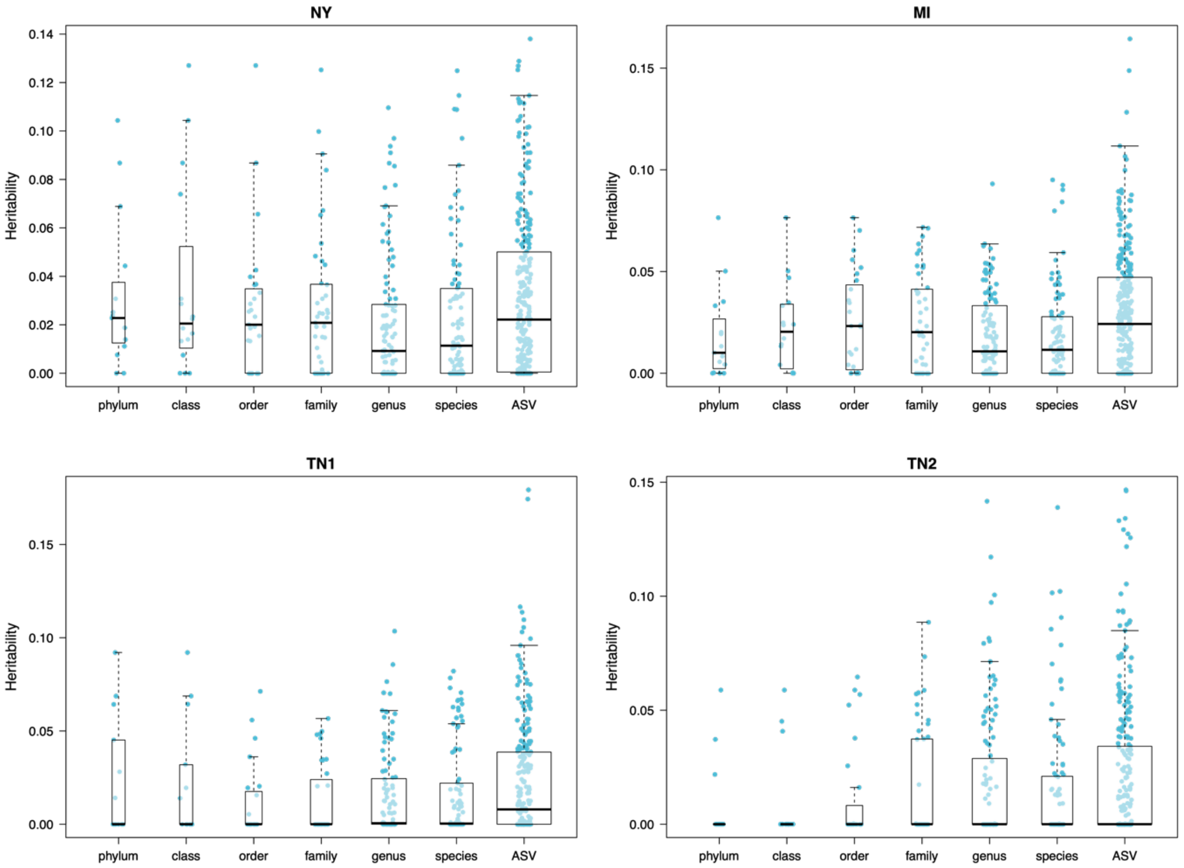

Supplementary Figure 3 Comparison of heritability at different taxonomic levels.

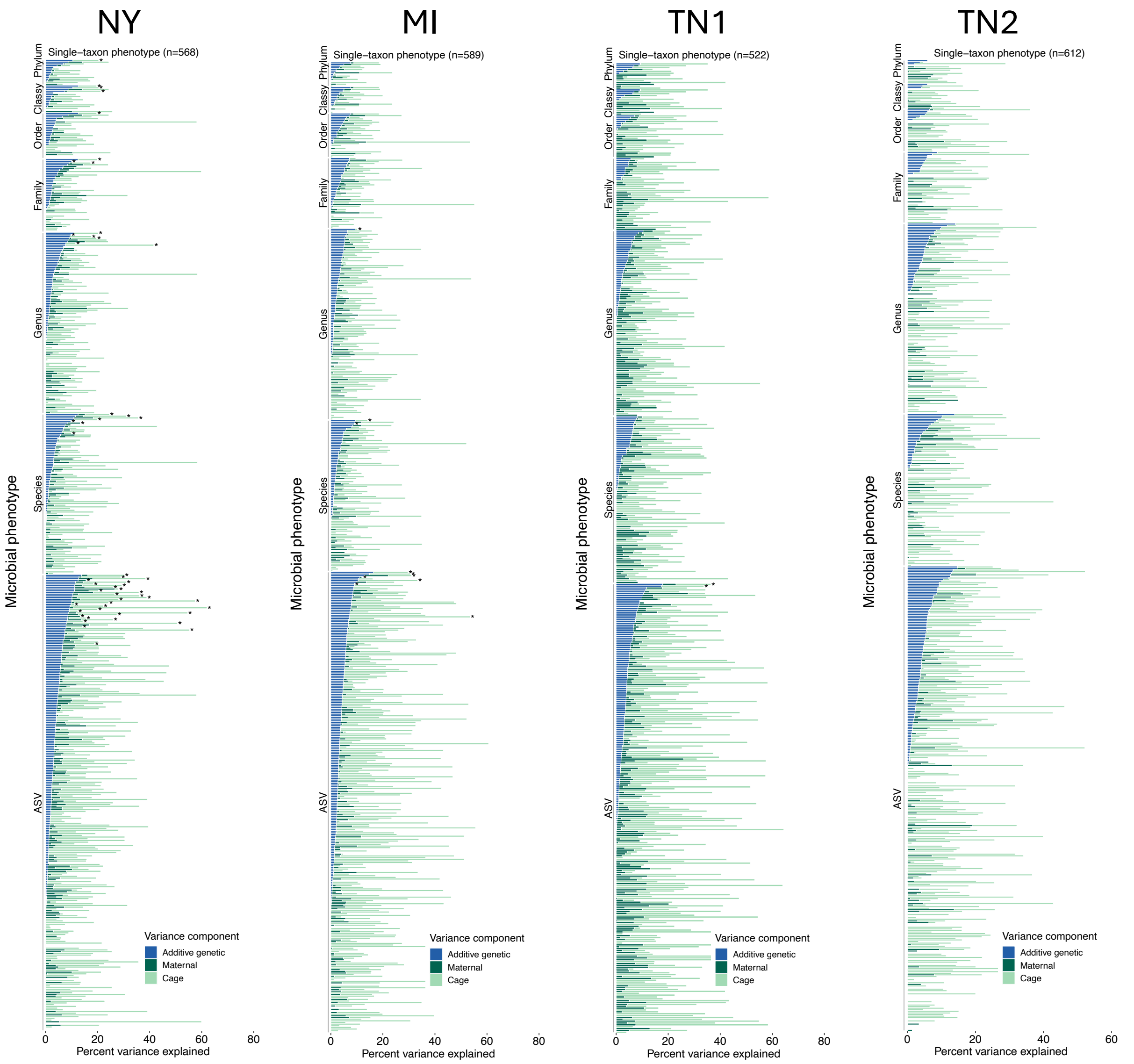

Supplementary Figure 4 Decomposition of the variance of microbiome phenotypes. Significant host genetic effects (cohort-wide FDR < 10%) are indicated with an asterisk. The code used to draw this figure is modified from the code provided by Grieneisen et al.[1].

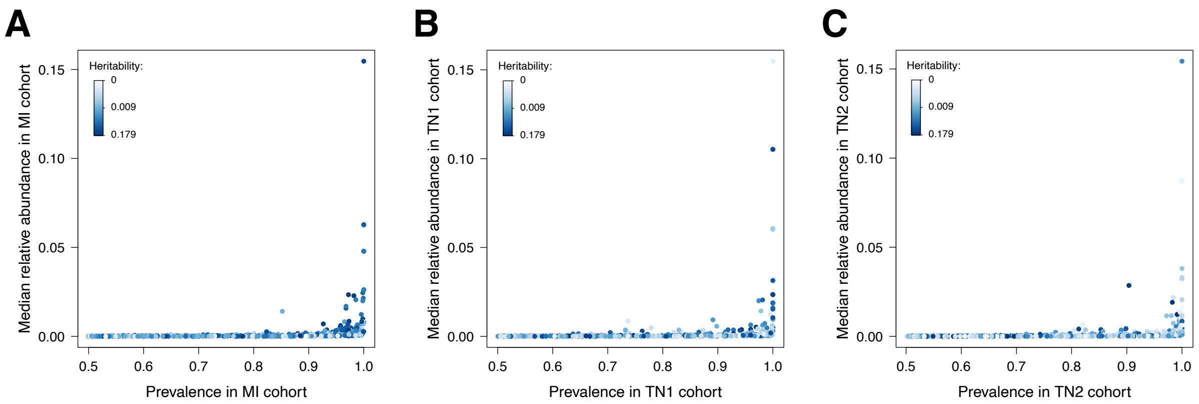

Supplementary Figure 5 Relationship between prevalence, relative abundance and heritability for the microbiome phenotypes measured in the MI, TN1, TN2 cohorts. The results for the NY cohorts are in main text (Fig. 3B).

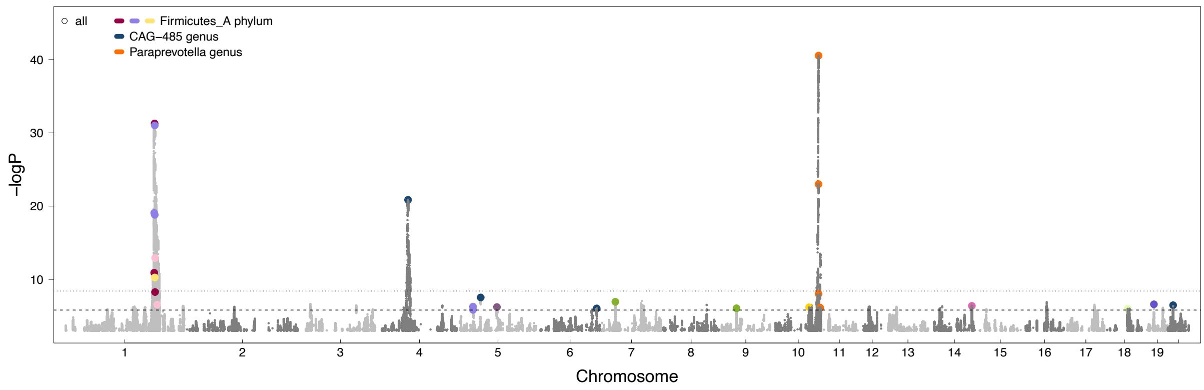

Supplementary Figure 6 Microbiome-associated loci in the whole sample (N = 3,784). This porcupine plot shows the association values for all 546 microbiome phenotypes with prevalence greater than 50% in the whole sample. The lower line (-logP = 5.8) reflects the genome-wide significance threshold for an individual trait, which accounts for the number of independent SNPs tested; the higher line (-logP=8.4) is the adjusted significance threshold, which in addition accounts for the number of independent microbiome phenotypes examined in each cohort. The colour of the dot refers to the genus affected; it is the same as in Fig.4. For that reason, genome-wide significant associations involving genera that did not have any genome-wide significant association in Fig. 4 are not higlighted by large coloured dots in this figure (but they are visible as grey peaks above the significance threshold).

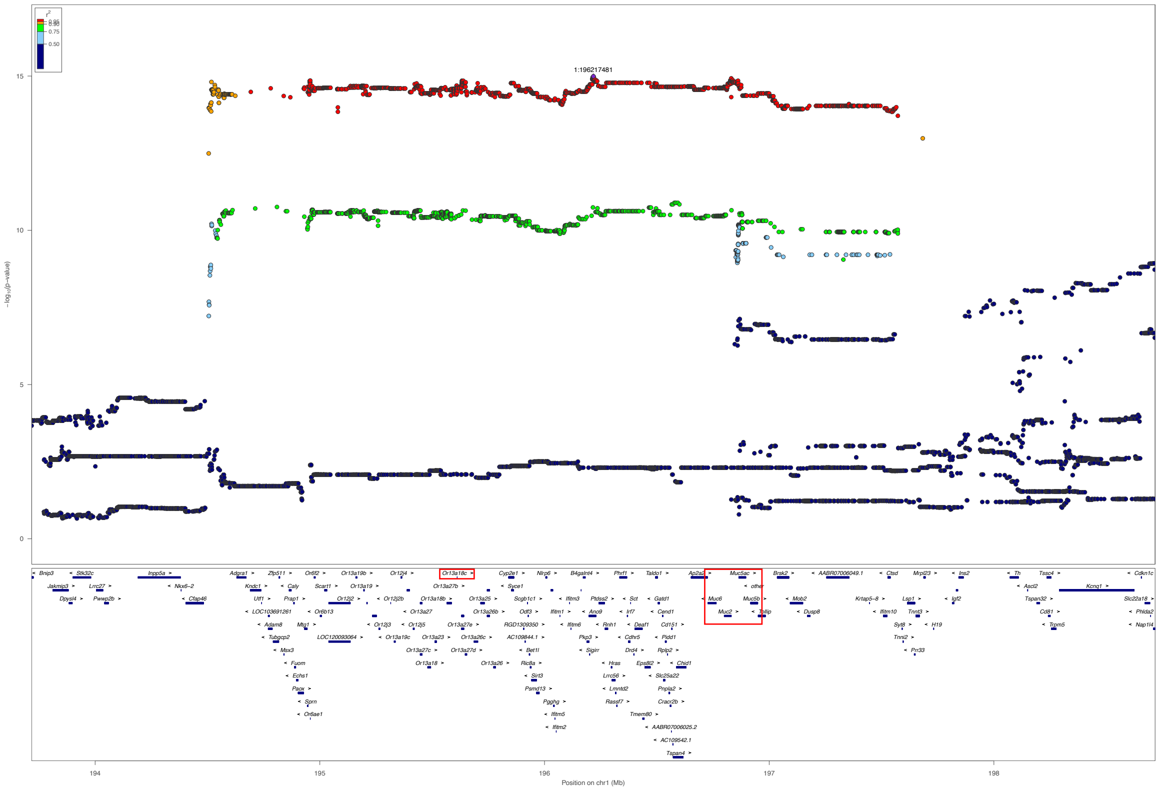

Supplementary Figure 7 Association (LocusZoom) plot for ASV_3613 (order TANB77, family CAG-508) measured in the NY cohort, which is the phenotype with the most significant association with the chromosome 1 replicated locus. The gene *Or13a18c* as well as the cluster of mucin-secreting genes (*Muc6, Muc2, Muc5ac and Muc5b*) are highlighted with red rectangles.

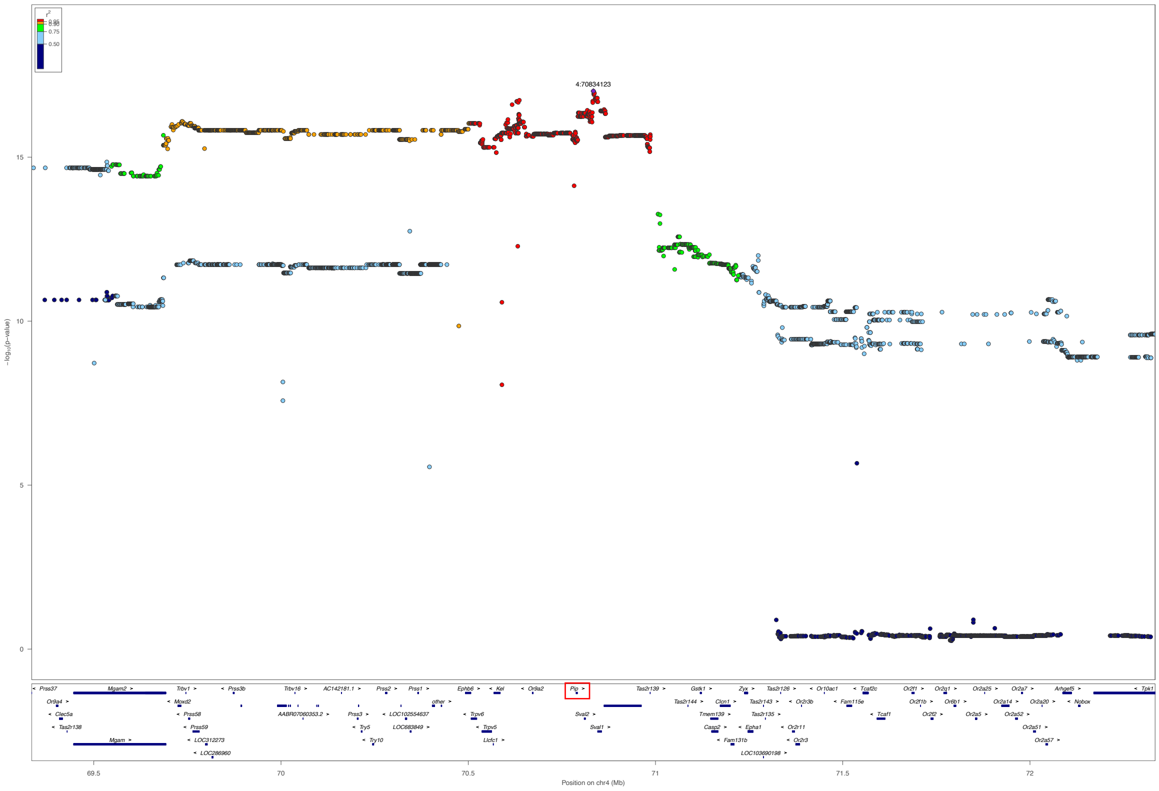

Supplementary Figure 8 Association plot for ASV_18566 (family Muribaculaceae, genus *CAG-485*, species *002362485*) measured in the NY cohort, which is the phenotype with the most significant association with the chromosome 4 replicated locus. The gene *Pip* is highlighted with a red rectangle.

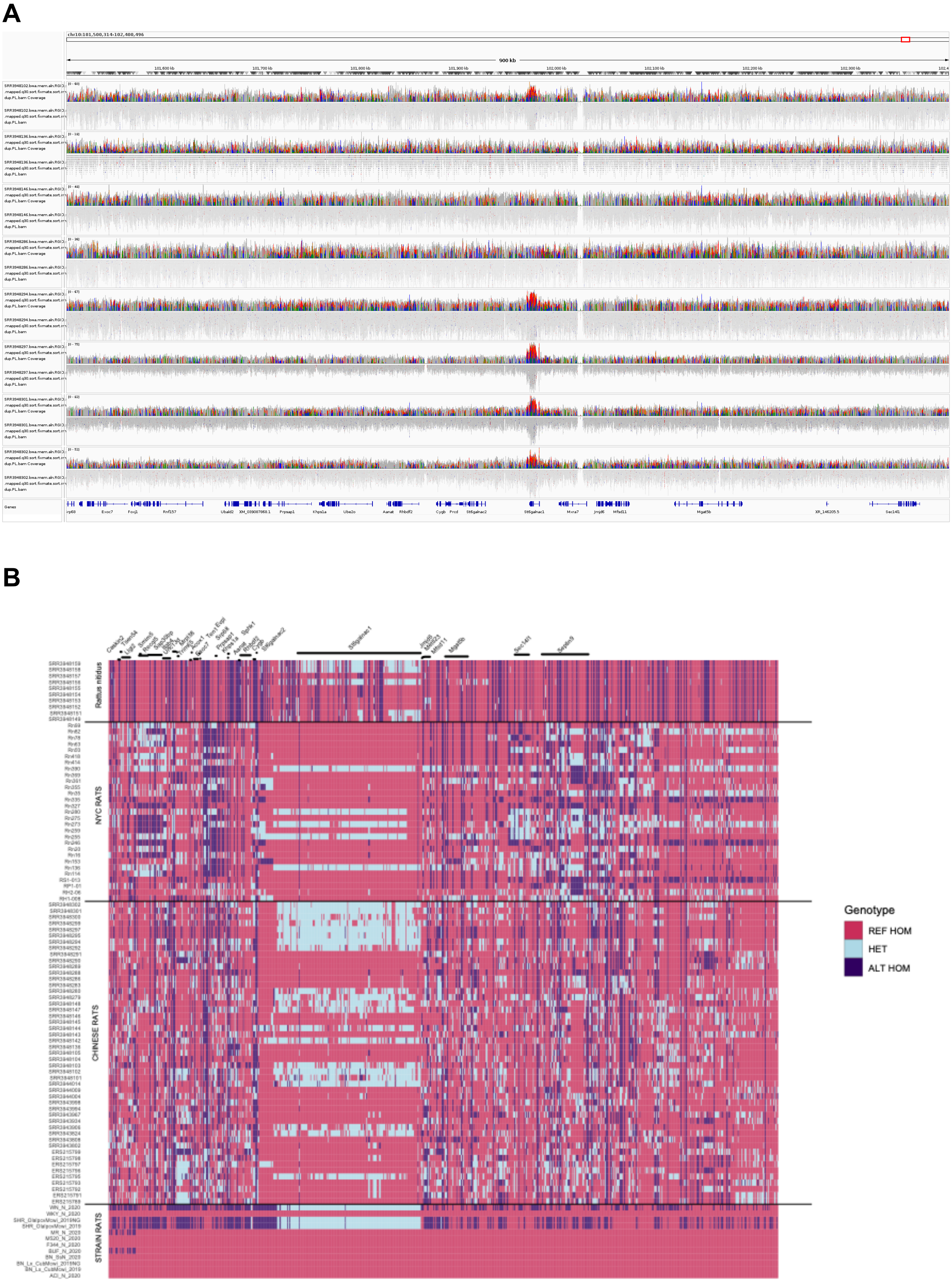

Supplementary figure 9 Evidence that the copy number variant affecting *St6galnac1* and seen in the WN/N HS founder and outbred HS rats (Fig. 5B) also segregates in wild rats. (A) Short read sequencing of wild rats captured in various locations in China and sequenced in Hiseq Ten X (data publicly available from Teng et

al. 2017[2]). The region surrounding the *St6galnac1* gene is shown. The bump in sequencing depth over *St6galnac1* shows that the same partial duplication/triplication of the gene segregates in wild rats from China as in the HS. (B) Genotypes of the eight HS founders (including WN/N) and two SHR inbred strains (bottom), wild rats from China (middle) and wild rats from New York City (VCF available from Harpak et al. 2020[3]) (top) showing a similar pattern of apparent variants accumulation in the region corresponding to the copy number variant. This apparent accumulation of variants is due to the presence of paralogous sequences, and results in apparent heterozygosity of the inbred strains.

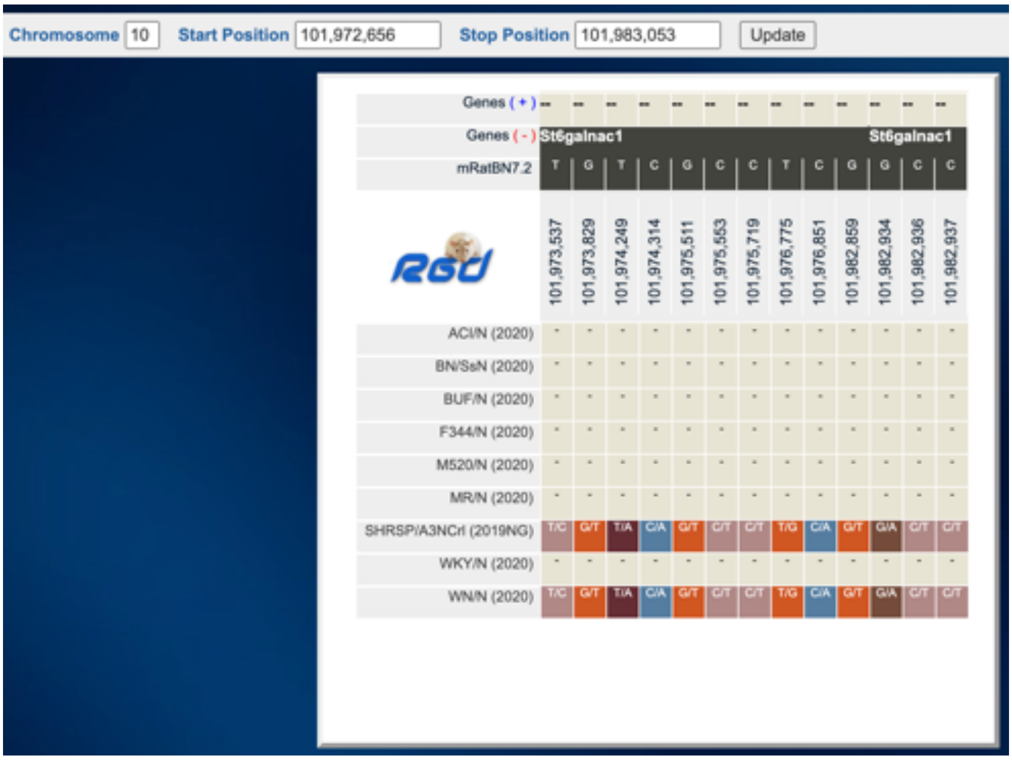

Supplementary figure 10 Screenshot from the Variant Visualizer of the Rat Genome Database[4] showing all variants identified in the eight HS founders and in the strain SHRSP/A3N (not an HS founder). This figure shows that WN/N and SHRSP/A3N are identical by descent in this region.

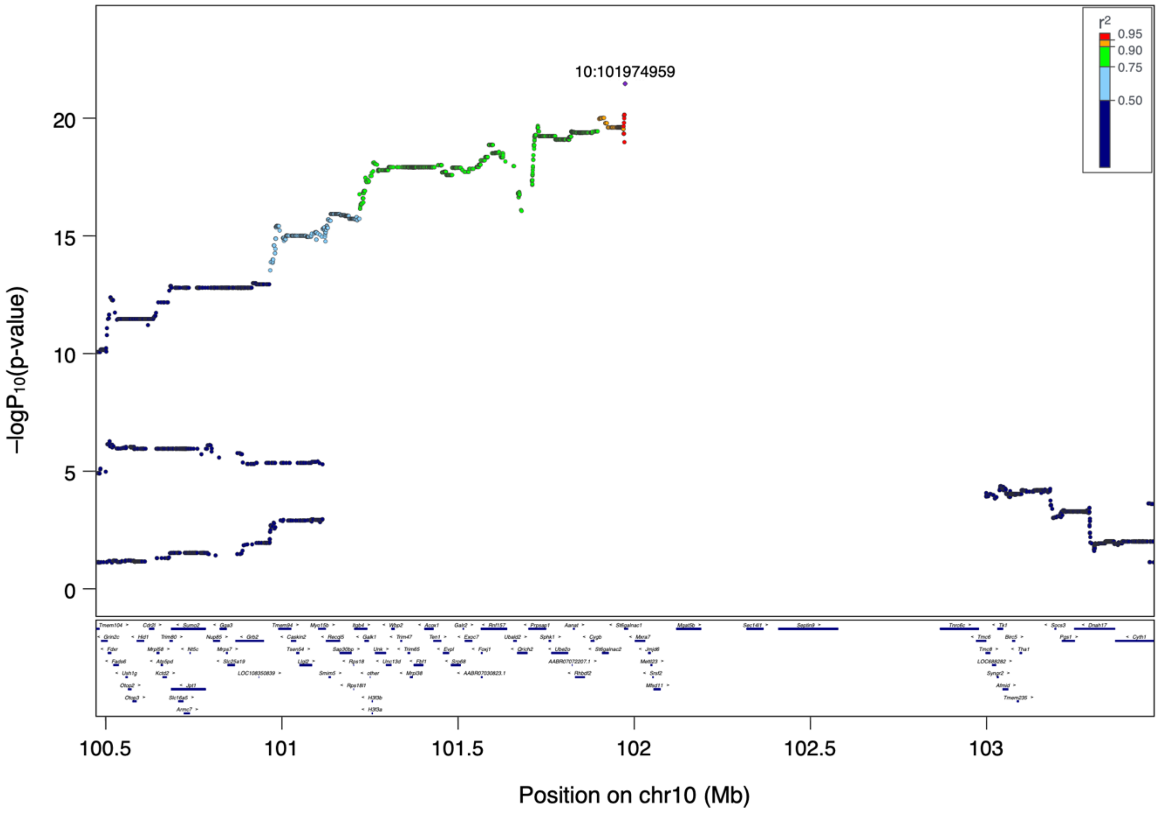

Supplementary figure 11. Local association plot for ASV_5163 (genus *Paraprevotella*) measured in the NY cohort on chromosome 10 (plot for zoomed in region is shown in Fig. 5A). The gap between 102-103Mb is not a technical issue: variants are detected between the eight inbred founders of the HS population but not in the outbred population. In that region, seven of the founders are identical by descent and the eighth founder (WN/N) is different. The WN/N haplotype was lost at some point during outbreeding, before reaching the generations included in this study.

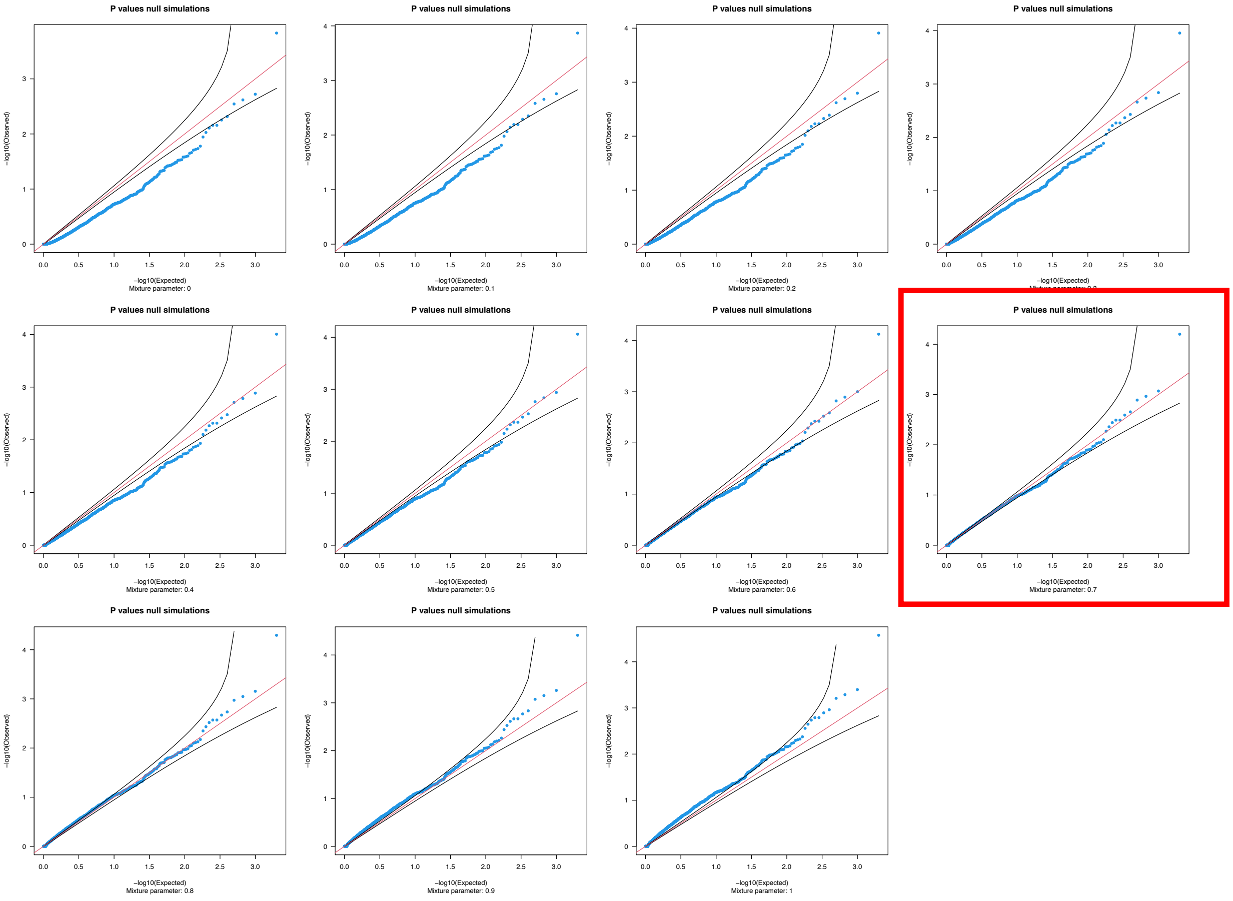

Supplementary Figure 12 Calibration of Mic-IGE *p*-values using null simulations. We chose 0.7 as the most appropriate parameter for the mixture of chi-square distributions with degrees of freedom 1 and 2 used to obtain Mic-IGE *p*-values. The resulting *p*-values obtained for null simulations are calibrated.

See Methods section “Evaluation of the calibration of *p*-values using null simulations” for full details.

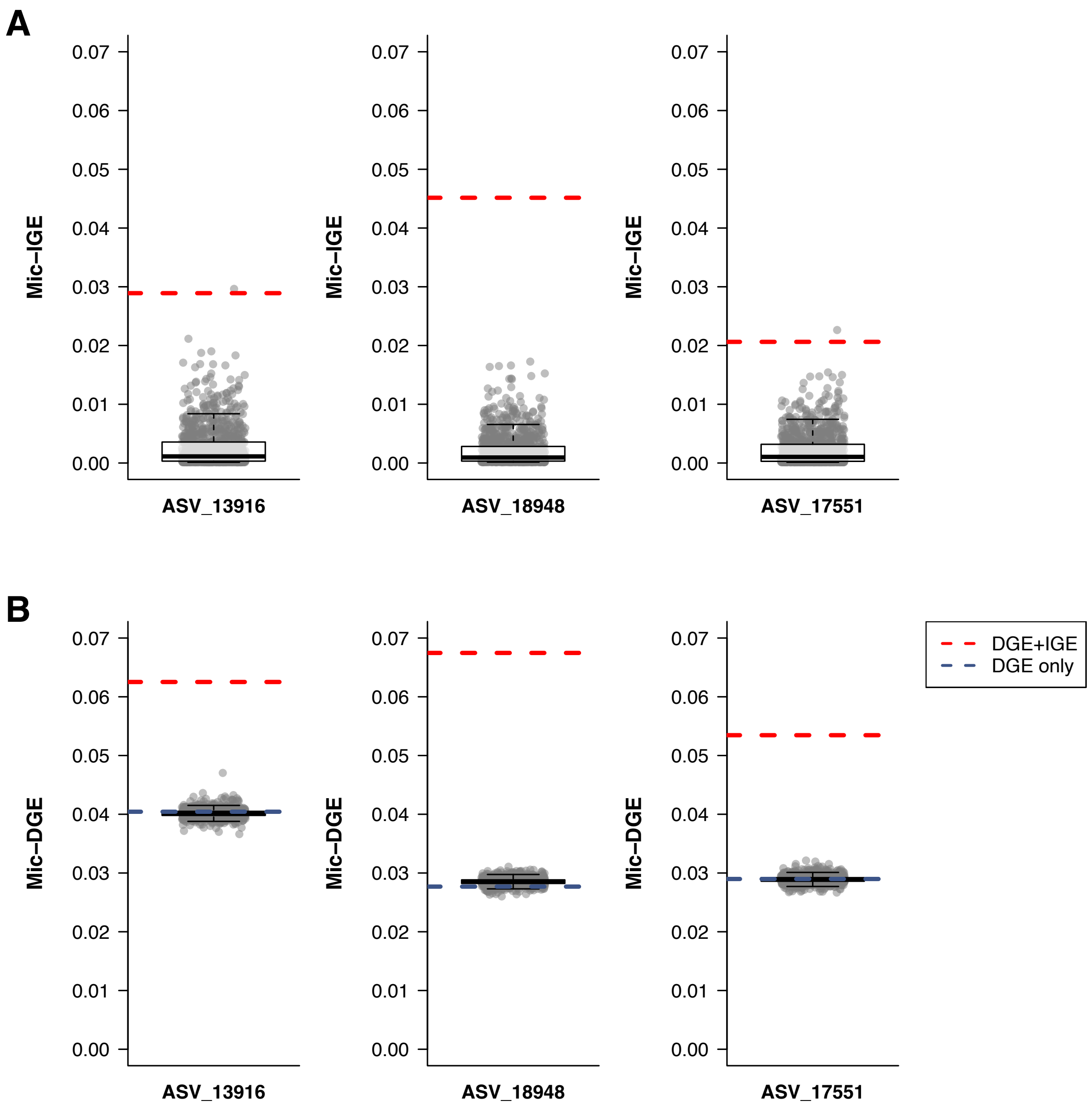

Supplementary Figure 13 Variance explained by Mic-IGE (A) and Mic-DGE (B) after permutation of cage mates' assignments. The cage assignments, used to account for cage effects, were not permuted.

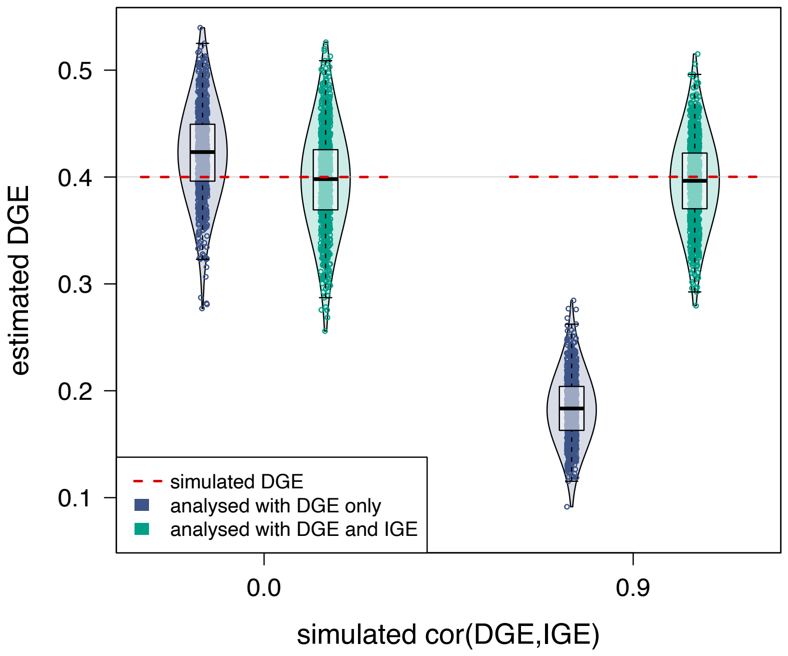

Supplementary Figure 14 Simulation study showing that DGE estimates are biased (underestimated) when IGE are at play but not modelled. Phenotypes were simulated for the rats in the MI cohort (see Figure 6D for simulations for the rats in the NY cohort, and Methods for details on the simulations).

	Cohort			
	NY	MI	TN1	TN2
**Facility rats were born in**	MCW	MCW	TN	MCW
**Facility rats were reared in, after weaning and shipment**	Univ. at Buffalo, NY	Univ. of Michigan, MI	Univ. of Tennessee, TN	Univ. of Tennessee, TN
**Number of rats per cage**	2	3	1-4	2
**Sexes of co-housed rats**	same-sex	same-sex	same-sex	one male, one female (breeding)
**Relatedness of co-housed rats**	unrelated	unrelated	littermates	unrelated
**Specific pathogen free facility**	yes	no	yes	yes
**Type of cages**	conventional	conventional	conventional & isolators	conventional & isolators
**Type of diet**	chow	chow	chow	chow
**Exact diet**	Teklad 18% Protein Rodent Diet (Envigo)	Picolab Laboratory Rodent Diet (LabDiet)	Teklad Irradiated LM-485 Mouse/Rat Diet (Envigo)	Teklad Irradiated LM-485 Mouse/Rat Diet (Envigo)
**Behavioural phenotyping***	locomotion, cocaine conditioning and behavioural regulations	cocaine sensitization and Pavlovian cue-reactivity	socially-acquired nicotine self-administration	none
**Drugs taken for phenotyping**	cocaine (intraperitoneal)	cocaine (intraperitoneal)	nicotine (intravascular)	none
**Age when ceca collected (months)**	6.5	3	2	6
**Overnight fasting prior to sacrifice**	yes	yes	no	yes
**Chemical used for euthanisia method**	isoflurane (inhalation)	none	isoflurane (inhalation)	isoflurane (inhalation)
**Physiological phenotyping (just before/after euthanasia)**	fasting glucose, adiposity	fasting glucose, adiposity	adiposity	fasting glucose, adiposity
**Cohort size (post filtering)**	1167	1112	950	555

* for a detailed description of the protocols used, see ratgenes.org, tab NIDA Research

Supplementary Table 1 Between-cohort environmental and experimental differences.

ASV or taxon	Cohort	Chr.	Position	-logP	Taxonomy (GTDB)
f__CAG-74	MI	1	196,695,784	9.9**	o__Christensenellales;f__CAG-74
ASV_59945	MI	1	196,831,050	7.6*	o__Christensenellales;f__CAG-74;g__Limiplasma
g__Limiplasma	MI	1	196,831,050	6.6*	o__Christensenellales;f__CAG-74;g__Limiplasma
s__Limiplasma_merdipullorum	MI	1	196,831,050	6.7*	o__Christensenellales;f__CAG-74;g__Limiplasma
ASV_63700	MI	1	195,080,343	9.0*	o__Christensenellales;f__CAG-74;g__Onthenecus
g__Onthenecus	MI	1	195,080,344	7.2*	o__Christensenellales;f__CAG-74;g__Onthenecus
ASV_70101	MI	1	196,498,032	5.8*	o__Oscillospirales;f__Oscillospiraceae_88309;g__Limivicinus
ASV_2972	MI	1	195,080,344	6.5*	o__TANB77;f__CAG-508;g__CAG-269
ASV_26581	NY	1	197,683,398	8.6**	o__TANB77;f__CAG-508;g__CAG-273
ASV_26581	TN2	1	196,878,658	6.0*	o__TANB77;f__CAG-508;g__CAG-273
ASV_29320	TN1	1	196,498,112	12.5**	o__TANB77;f__CAG-508;g__CAG-793
ASV_29320	MI	1	195,701,712	6.3*	o__TANB77;f__CAG-508;g__CAG-793
g__CAG-793	NY	1	195,629,919	7.5*	o__TANB77;f__CAG-508;g__CAG-793
s__CAG-793_sp000433915	NY	1	195,629,919	9.5*	o__TANB77;f__CAG-508;g__CAG-793
**ASV_3613**	**NY**	**1**	**196,217,481**	**15.0****	**o__TANB77;f__CAG-508;g__UMGS1994**
ASV_3613	MI	1	197,534,412	10.6**	o__TANB77;f__CAG-508;g__UMGS1994
ASV_3613	TN2	1	197,572,275	6.9*	o__TANB77;f__CAG-508;g__UMGS1994
g__UMGS1994	TN1	1	196,916,401	6.9*	o__TANB77;f__CAG-508;g__UMGS1994
g__UMGS1994	MI	1	197,208,428	6.4*	o__TANB77;f__CAG-508;g__UMGS1994
s__UMGS1994_sp900553945	MI	1	197,208,428	7.0*	o__TANB77;f__CAG-508;g__UMGS1994
s__UMGS1994_sp900553945	TN1	1	196,916,401	6.6*	o__TANB77;f__CAG-508;g__UMGS1994

Supplementary Table 2 Significant and suggestive associations at the chromosome 1 replicated locus. Genome-wide significance (-logP > 5.8) is shown with one asterisk (*). Associations significant after accounting for the number of microbiome phenotypes tested in each cohort (Bonferroni correction, -logP > 8.4) are shown with two asterisks (**). The lead association is shown in bold. Only associations in strong LD with it (R^2 > 0.8) are included in the table. The associations are coloured based on the colour they appear in in Fig. 3. The first row refers to a microbiome phenotype that does not appear in the porcupine plot because it is only annotated at the family level, not at the genus level, hence it appears in white (no colour). All these ASVs and taxa belong to the Firmicutes_A phylum (GTDB taxonomy).

ASV or taxon	Cohort	Chr.	Position	-logP	Taxonomy (GTDB)
**ASV_18566**	**NY**	**4**	**70,834,123**	**17.0****	**o__Bacteroidales;f__Muribaculaceae;g__CAG-485**
ASV_18566	MI	4	70,349,586	7.0*	o__Bacteroidales;f__Muribaculaceae;g__CAG-485
ASV_18566	TN1	4	70,473,002	5.9*	o__Bacteroidales;f__Muribaculaceae;g__CAG-485

Supplementary Table 3 Significant and suggestive associations at the chromosome 4 replicated locus. Genome-wide significance (-logP > 5.8) is shown with one asterisk (*). Associations significant after accounting for the number of microbiome phenotypes tested in each cohort (Bonferroni correction, -logP > 8.4) are shown with two asterisks (**). The lead association is shown in bold. Only associations in strong LD with it ($R^2 > 0.8$) are included in the table. The associations shown in blue are those that appear in blue in Fig. 3.

ASV or taxon	Cohort	Chr.	Position	-logP	Taxonomy (GTDB)	Direction of effect
ASV_5095	MI	10	101,922,691	10.4**	o__Bacteroidales;f__Bacteroidaceae;g__Paraprevotella	WN/N decreasing
**ASV_5163**	**NY**	**10**	**101,974,959**	**21.5****	**o__Bacteroidales;f__Bacteroidaceae;g__Paraprevotella**	**WN/N decreasing**
ASV_5163	TN1	10	101,972,949	8.5**	o__Bacteroidales;f__Bacteroidaceae;g__Paraprevotella	WN/N decreasing
ASV_5163	TN2	10	101,769,054	4.2	o__Bacteroidales;f__Bacteroidaceae;g__Paraprevotella	WN/N decreasing
g__Paraprevotella	NY	10	101,727,325	12.5**	o__Bacteroidales;f__Bacteroidaceae;g__Paraprevotella	WN/N decreasing
g__Paraprevotella	MI	10	101,938,835	10.3**	o__Bacteroidales;f__Bacteroidaceae;g__Paraprevotella	WN/N decreasing
g__Paraprevotella	TN2	10	101,905,255	4.1	o__Bacteroidales;f__Bacteroidaceae;g__Paraprevotella	WN/N decreasing
ASV_2821	MI	10	101,974,959	4.0	o__Verrucomicrobiales;f__Akkermansiaceae;g__Akkermansia	WN/N decreasing
ASV_17008	MI	10	101,972,949	4.5	o__Bacteroidales;f__Muribaculaceae;g__Muribaculum	WN/N increasing

Supplementary Table 4 Significant and suggestive associations at the chromosome 10 replicated locus. Genome-wide significance (-logP > 5.8) is shown with one asterisk (*). Associations significant after accounting for the number of microbiome phenotypes tested in each cohort (Bonferroni correction, -logP > 8.4) are shown with two asterisks (**). The lead association is shown in bold. Only associations in strong LD with it (R^2 > 0.8) are included in the table. The associations shown in orange are those that appear in orange in Fig. 3.

SUPPLEMENTARY REFERENCES

1. Grieneisen, L. *et al.* Gut microbiome heritability is nearly universal but environmentally contingent. *Science* **373**, 181-186 (2021).
2. Teng, H. *et al.* Population genomics reveals speciation and introgression between brown Norway rats and their sibling species. *Molecular Biology and Evolution* **34**, 2214-2228 (2017).
3. Harpak, A. *et al.* Genetic Adaptation in New York City Rats. *Genome Biology and Evolution* **13**(2020).
4. Rat Genome Database Web Site, M.C.o.W., Milwaukee, Wisconsin. Variant Visualizer.
